# Antimicrobial Drugs in Fighting against Antimicrobial Resistance

**DOI:** 10.3389/fmicb.2016.00470

**Published:** 2016-04-08

**Authors:** Guyue Cheng, Menghong Dai, Saeed Ahmed, Haihong Hao, Xu Wang, Zonghui Yuan

**Affiliations:** ^1^Ministry of Agriculture Laboratory for Risk Assessment of Quality and Safety of Livestock and Poultry Products, Huazhong Agricultural UniversityWuhan, China; ^2^National Reference Laboratory of Veterinary Drug Residues (HZAU) and Ministry of Agriculture Key Laboratory for the Detection of Veterinary Drug Residues in Foods, Huazhong Agricultural UniversityWuhan, China

**Keywords:** antimicrobial resistance, biofilm, persisters, antimicrobial drug, nanoparticles

## Abstract

The outbreak of antimicrobial resistance, together with the lack of newly developed antimicrobial drugs, represents an alarming signal for both human and animal healthcare worldwide. Selection of rational dosage regimens for traditional antimicrobial drugs based on pharmacokinetic/pharmacodynamic principles as well as development of novel antimicrobials targeting new bacterial targets or resistance mechanisms are key approaches in tackling AMR. In addition to the cellular level resistance (i.e., mutation and horizontal gene transfer of resistance determinants), the community level resistance (i.e., bilofilms and persisters) is also an issue causing antimicrobial therapy difficulties. Therefore, anti-resistance and antibiofilm strategies have currently become research hotspot to combat antimicrobial resistance. Although metallic nanoparticles can both kill bacteria and inhibit biofilm formation, the toxicity is still a big challenge for their clinical applications. In conclusion, rational use of the existing antimicrobials and combinational use of new strategies fighting against antimicrobial resistance are powerful warranties to preserve potent antimicrobial drugs for both humans and animals.

## Introduction

Antimicrobial resistance (AMR) is one of the ultimate fears to the health of humans and animals worldwide. Use of antimicrobial drugs in humans or animals results in the emergence and dissemination of resistant bacteria, and overuse or abuse of them makes this situation worse. Thus, it is important to simultaneously preserve effective antimicrobials as long as possible as well as continue to employ them for the service of human and animal health (Chang et al., 2015).

The dissemination of AMR has not been paralleled by the development of novel antimicrobials. This is due to that the process of drug discovery and clinical trials of new antimicrobials takes a long time, and only a few new agents have recently been approved for use. These situations prompt the efforts to develop alternatives to traditional antimicrobials, as described in our previous review (Cheng et al., 2014). However, some of the alternatives are only used for the prevention of bacterial infections (e.g., vaccines); some show indirect effect against pathogens (e.g., immunomodulators, feed enzymes); some are of complex composition (e.g., probiotics, plant extracts), thus the effects vary greatly; and the antimicrobial peptides, such as one of the bacteriocins, lantibiotics, have been reported causing bacterial resistance (Draper et al., 2015).

In this review, we briefly focus on old and novel antimicrobial agents in tackling AMR. The AMR occurs on two levels, the cellular level resistance (mutation and horizontal gene transfer (HGT) of resistance determinants) and the community level resistance (biofilm and persister cells) (Penesyan et al., 2015). The studies reviewed suggest that only rational use of existing old antimicrobial drugs and combinational use of anti-resistance or antibiofilm strategies with antimicrobials as well as continuing development of new antimicrobial agents could fight against AMR.

## Mechanisms of AMR

AMR includes two levels of resistance, the cellular level resistance and the community level resistance (Penesyan et al., 2015; Table 1). The development of cellular resistance occurs due to endogenous gene mutations as well as via HGT of resistance determinants from other microorganisms. Also, a group of bacteria can be tolerant to the environmental stress that individual cells cannot, which is called the community level resistance. Such tolerance can cause an increased resistance to antimicrobials (Penesyan et al., 2015). For example, the resistance obtained by microorganisms in biofilm can be up to 1000 times higher than that gained by their planktonic counterparts, which impairs the treatment of biofilm-associated infections in clinical treatment (Lebeaux et al., 2014b; Penesyan et al., 2015). The main mechanism currently proposed to explain such tolerance is the presence of persister cells. The persisters can escape the lethal action of antimicrobials by entering a physiological state in which the antimicrobials do not kill them, a phenomenon known as bacterial persistence (Maisonneuve and Gerdes, 2014). Moreover, the cellular and community levels of resistance can be synergistic, thereby greatly enhancing the overall AMR of the microbial community (Penesyan et al., 2015).

**Table 1:** **Mechanisms of AMR (derived from Penesyan et al., 2015)**.

**Mechanisms**	**References**
**CELLULAR LEVEL RESISTANCE**	
Drug inactivation by hydrolysis (e.g., β-lactamase for β-lactam resistance) or modification (e.g., acetyltransferases for aminoglycoside resistance)	Shaw et al., 1993; Bush and Fisher, 2011
Target alteration by reducing the binding affinity to the drug (e.g., DNA gyrase mutation for fluoroquinolone resistance) or bypassing the drug target	Hooper and Jacoby, 2015
Reducing drug influx by decreased permeability (e.g., the Gram-negative outer membrane)	Nikaido, 2003
Drug extrusion via efflux pumps	Li et al., 2015
HGT of resistance determinants from other microorganisms	D'Costa et al., 2006
**COMMUNITY LEVEL RESISTANCE**	
Biofilm matrix acts as a shield against antimicrobials (e.g., polysaccharides against aminoglycoside, extracellular DNA against cationic peptides)	Mulcahy et al., 2008; Khan et al., 2010; Chiang et al., 2013
Antimicrobials targeting dividing bacteria have a limited effect against the slow or non-growing persisters	Lewis, 2008, 2010
Starvation-induced stringent response caused by nutrient limitation in biofilm mediates high biofilm-specific resistance	Nguyen et al., 2011; Bernier et al., 2013
**SYNERGY BETWEEN COMMUNITY AND CELLULAR LEVEL RESISTANCE**	
Significantly enhanced mutation rate in biofilms leads to faster development of resistant mutants	Conibear et al., 2009
Extracellular DNA in biofilm facilitates HGT of resistance determinants, encourages the acquisition and exchange of resistant integron gene cassettes, and promotes conjugation and natural transformation	Blázquez, 2003; Townsend et al., 2003; Gillings et al., 2009; Domingues et al., 2012
The sub-inhibitory concentration of antimicrobials in biofilm is favorable to increasing the rates of mutation, recombination and HGT	Gillings and Stokes, 2012; Morita et al., 2014
Mechanisms of cellular level resistance can act in a biofilm-specific manner	Zhang and Mah, 2008

## Revisit to old antimicrobials

As AMR to commonly used antimicrobial drugs increases, older antimicrobials are being “revived” and attracting attention. These old antimicrobials represent a new alternative for the control of AMR (Pulcini et al., 2012). Cassir et al. (2014) have provided a collection of microbiological and clinical data on potentially useful older antimicrobials for the successful treatment of multidrug-resistant (MDR) Gram-negative bacterial infections (e.g., polymyxins, fosfomycin, mecillinam, temocillin, and nitrofurantoin), MDR Gram-positive infections (e.g., trimethoprim-sulfamethoxazole, tetracyclines, chloramphenicol, clindamycin, pristinamycin, rifampicin, and fusidic acid), and MDR tuberculosis (e.g., clofazimine, amoxicilline-clavulanate, trimethoprim-sulfamethoxazole, and minocycline). Since older antimicrobials have rarely been subjected to present drug development procedures, they are less considered in practice guidelines. Therefore, their efficacy and safety must be revaluated to optimize therapy.

A number of antimicrobials discovered decades ago that have unique chemical scaffolds and/or utilize novel modes of action to interact with bacterial targets, such as ribosome (Arenz and Wilson, 2016). For example, dityromycin, a cyclic decapeptide antibiotic produced by *Streptomyces* sp., can uniquely bind to ribosomal protein S12 in the small ribosomal subunit, a mode of action different than any other known translational inhibitor (Bulkley et al., 2014). In many cases, these “forgotten” compounds display cytotoxicity against eukaryotic cells and thus were abandoned (Arenz and Wilson, 2016). However, recent structure-function analysis gives us better understanding of the similarities and differences between bacterial targets and their eukaryotic counterparts, thereby guiding the future development of more specific and less toxic inhibitors. With the increased understanding of AMR mechanisms, revisiting the known antimicrobials are helpful to the exploration of the next generation of antimicrobial drugs.

Procedures for registration of antimicrobials drugs have improved significantly. Both EU (EMA, 2013) and US authorities (FDA, 2010) have published numerous guidance documents, and addressed the increasing need for antimicrobials that are active against MDR bacteria. The guidance documents include recommendations for dosage regimens based on pharmacokinetic (PK)/pharmacodynamic (PD) relationships. PK/PD provides a universal framework for exposure-response relationships, and the responses include efficacy, toxicity, and emergence of resistance (Muller et al., 2015). Exposure-response relationships also provide a means to translate experimental and preclinical data into the clinical settings, including setting clinical breakpoints, as extensively described by the European Committee on Antimicrobial Susceptibility Testing (EUCAST) (Mouton et al., 2012). To determine the optimal dose, several key features of the exposure-response relationship need to be determined, including MIC distribution of the interested microorganisms, the PK profiles for a variety of doses and patient populations, and the exposure-response relationship and PD target (Muller et al., 2015).

There are knowledge gaps for those revived antimicrobials in the areas of PK profiling in patients, as well as PD targets derived from preclinical and clinical studies (Muller et al., 2015). Although the regulatory requirements for new antimicrobial agents have become more and more rigorous, updates of the product information for old antimicrobials are either missing or insufficient, which would pose significant risks of potential harm to the patients. In addition, there is no motivation for companies to develop antimicrobials when the cost and time of drug approval is far beyond commercial interests, even if there is an obvious medical need.

In summary, redevelopment of an old antimicrobials leads to an improved understanding of its chemistry, PK/PD as well as optimizing its clinical use in different patient populations. Optimization of antimicrobial therapy in terms of PK/PD is essential to improve therapeutic efficacy but minimize the toxicity and the risk of resistance development during treatment (Mouton et al., 2011). As old antimicrobials are rarely included in surveillance programs, the evaluation of the risks of drug resistance is lacking. The prescription of old antimicrobials needs to be regulated by professional antimicrobial stewardships. Besides, as public health concern, cost effectiveness should be integrated in further comparisons between old and currently used antimicrobials.

## Development of new antimicrobials

The current antimicrobials, mainly derived from natural sources, inhibit cellular processes such as cell wall biosynthesis, DNA replication, and protein synthesis. With the worldwide emergence of AMR, there is renewed interest in the investigation of alternative essential cellular processes, including bacterial central metabolic pathways, as the drug targets for the next generation of antimicrobials (Murima et al., 2014). For examples, bedaquiline is an antitubercular drug targeting the F_0_F_1_ ATP synthase (Andries et al., 2005). Like bedaquiline, Q203, an optimized imidazopyridine amide compound, selectively inhibits the respiratory cytochrome *bc1* complex in mycobacteria regardless of architectural conservation of the *bc1* complex in many species (Pethe et al., 2013). The inhibition of the bacterial divisome, mainly by targeting the central cell division mediator FtsZ, has been accepted as a promising strategy for antimicrobial attack by either interfering with the natural dynamics and functions of FtsZ during the cell cycle or activating a bacterial protease to degrade FtsZ, thus causing bacterial death in a suicidal manner (Sass and Brötz-Oesterhelt, 2013). The mode of action of alkyl gallate is a combination of direct targeting of FtsZ and permeabilization of bacterial membranes, which is a promising hit for the further development of antibacterials (Król et al., 2015). Recent efforts have also been devoted to developing drugs that interrupt the assimilation of iron by bacteria, a process that is vital to cellular homeostasis (Foley and Simeonov, 2012). The unique asymmetric outer membrane in Gram-negative bacteria, which acts as a permeability barrier that protects the cell from external stresses such as the presence of antimicrobials, has become an attractive drug target. A novel β-hairpin macrocyclic peptide, JB-95, exhibits an ability to selectively disrupt the outer membrane through interactions with selected β-barrel outer membrane proteins including BamA and LptD, but not the inner membrane of *E. coli* (Urfer et al., 2016). Furthermore, the bacterial protein secretion pathway is a target for eliminating or disarming pathogens. Targeting the Sec-pathway for novel antimicrobial agents focuses on two key components: SecA, the ATP-driven motor protein responsible for driving preproteins across the cytoplasmic membrane and the Type I signal peptidase which is responsible for the removal of the signal peptide to allow the release of the mature protein from the membrane (Rao et al., 2014).

Targeting resistance, usually used in combination with the traditional antimicrobials, is another strategy to fight against AMR. Among the four general resistance mechanisms, which include target alteration (Hooper and Jacoby, 2015), drug inactivation (Shaw et al., 1993; Bush and Fisher, 2011), decreased permeability (Nikaido, 2003) and increased efflux (Sun et al., 2014; Li et al., 2015), drug extrusion by multidrug efflux pumps serves as an important mechanism of MDR. Efflux pumps also have physiological functions in response to various of environmental and physiological signals (Sun et al., 2014). Recent studies have tried to reverse the resistance phenotype conferred by efflux pump activation (Opperman and Nguyen, 2015; Venter et al., 2015). It was observed that the addition of efflux pump inhibitors partially restored drug susceptibility *in vitro* and *in vivo* in the anti-*Mycobacterium tuberculosis* therapy (Pule et al., 2016). The class of zinc-dependent hydrolases, metallo-β-lactamase, can confer bacteria with extended spectrum β-lactam resistance. The design of compounds with the β-lactam core scaffold is an attractive approach to the development of metallo-β-lactamase inhibitors. Some promising inhibitors, including cephalosporin derivatives, penicillin derivatives, carbapenem derivatives, cyclobutanone β-lactam analogs, thiol derivatives, succinic acid derivatives, mercaptoacetic acid thioester derivatives, pyrrole-, pyridine- and triazole-containing compounds, and DNA aptamer, have been thoroughly reviewed by King and Strynadka (King and Strynadka, 2013). Targeting the resistance mechanism itself by a vaccine is an interesting but hitherto unexplored approach (Henriques-Normark and Normark, 2014). Vaccination directed against the resistance mechanism can be possible when resistance is mediated by an enzyme whose activity can be inhibited by neutralizing antibodies.

Except for the above inhibitors targeting resistance, drugs in already-known classes such as new β-lactams, quinolones, aminoglycosides, and tetracyclines have been designed to escape from many of the known resistance mechanisms. BAL30072, a siderophore monosulfactam similar to aztreonam, exhibits antibacterial activity against most species of aerobic Gram-negative bacteria (Page et al., 2010). It is stable toward metallo-β-lactamases and is a poor substrate for many serine carbapenemases. Several new quinolones, such as avarofloxacin, delafloxacin, finafloxacin and the desfluoroquinolone nemonoxacin, which show enhanced activity against fluoroquinolone-resistant Gram-positive bacteria including MRSA are in clinical development (Page and Bush, 2014). A modified aminoglycoside, plazomicin, has been demonstrated activity against both Gram-negative and Gram-positive bacterial pathogens (Zhanel et al., 2012). Modified tetracyclines, such as tigecycline, omadacycline and eravacycline are of interest for their activity against many MDR *Enterobacteriaceae* and *Acinetobacter* spp., including isolates expressing tetracycline-specific efflux and ribosomal protection proteins (Sutcliffe, 2011).

## Approaches to combat biofilms

The approaches to combat biofilms are extensively reviewed by Beloin et al. (2014) (Table 2). During the biofilm development, the bacteria initially adhere to a surface that ultimately leads to colonization and infection by pathogenic bacteria. Therefore, reducing adhesion is a strategy to prevent biofilm formation and related infections (Veerachamy et al., 2014). Recently, non-specific inhibition of adhesion vs. targeting specific adhesions has been developed to reduce bacterial adhesion. Quorum sensing (QS), which controls many important physiological processes such as biofilm development, is a widespread intercellular form of communicating and cooperative activities of bacteria at the population level, and it depends on the production, secretion, and detection of small diffusible autoinducers, such as acyl-homoserine lactones, auto-inducing peptides and autoinducer 2 (Zhang and Li, 2016). Cyclic di-GMP is a second messenger that acts to regulate a wide range of functions including developmental transitions, adhesion and biofilm formation (Caly et al., 2015). Targeting these signaling pathways is also a strategy to prevent biofilm development. Moreover, using enzymes or chelating agents can hydrolyze biofilm components or destabilize biofilm matrix. On the other hand, persister cells have recently been subjected to an intensive research in order to limit biofilm-associated antimicrobial tolerance. The formation of persister cells depends on the ubiquitous bacterial regulatory nucleotides tetra and penta-guanosine phosphate (ppGpp) that activate inhibitors of cell growth (Germain et al., 2015). Therefore, interfering ppGpp could inhibit the formation of persister cells.

**Table 2:** **Recent studies of some promising antibiofilm strategies**.

**Mode of actions**	**Categories**	**Reported approaches**	**References**
Non-specific anti-adhesion	Anti-adhesive polymers	Polymers comprising ester and cyclic hydrocarbon moieties reduced the attachment of *E. coli, P. aeruginosa* and *S. aureus*	Hook et al., 2012
		Vascular catheters with a non-leaching poly-sulfobetaine surface modification reduced the adhesion of *E. coli* and *S. aureus*	Smith et al., 2012
		Methyl-cellulose-coated totally implantable venous access ports implanted in rats inhibited the adhesion of *P. aeruginosa* and *S. aureus*	Chauhan et al., 2014
		Dental adhesives containing dimethylaminododecyl methacrylate inhibited *S. mutans, S. gordonii*, and *S. sanguinis* multispecies biofilms	Zhang et al., 2015
Specific anti-adhesion	Impeding adhesion biogenesis	AL1 targeted the subunit polymerization of the type 1 pilus assembly, thus disrupted the pilus-dependent biofilm formation in uropathogenic *E. coli*.	Lo et al., 2014
	Lectins competitors	Inhibitors of the type 1 fimbriae adhesion prevented catheter-associated urinary tract infections and chronic cystitis in mice infected by *E. coli*	Totsika et al., 2013
	Targeting virulence factor	Limonene showed antibiofilm activity against *S. pyogenes, S. mutans* and *S. mitis* by downregulation of the surface-associated virulence factor genes	Subramenium et al., 2015
	Bulky hydrocarbons	Maltose derivatives with bulky hydro-carbon groups exhibited an inhibition of adhesins/receptors mediated binding of *P. aeruginosa*	Shetye et al., 2014
Targeting signaling pathways	Interfering cyclic-di-GMP	Inhibitors of diguanylate cyclase enzymes that synthesize cyclic-di-GMP inhibited the biofilm formation by *Vibrio cholerae*	Sambanthamoorthy et al., 2012
	Interfering QS	Analogs of QS autoinducers or enzymes degrading QS molecules were used to quench QS-controlling biofilm formation	Zhu and Kaufmann, 2013; Rampioni et al., 2014
		Inhibitors of the quorum regulator, the staphylococcal accessory regulator A (SarA), showed antibiofilm activity against *S. aureus*	Balamurugan et al., 2015
Dispersing biofilm matrix	Enzymes	Degradation of extracellular DNA by DNaseI or hydrolyzing poly-*N*-acetylglucosamine by Dispersin B dispersed biofilms *in vitro* and *in vivo*	Darouiche et al., 2009; Hymes et al., 2013
		Phage depolymerases can degrade the extracellular polymers to allow the permeation of bacteriophages into deeper biofilm layers to kill bacteria	Parasion et al., 2014
	Chelating agents	EDTA was an efficient adjuvant to gentamicin to eradicate *E. coli, P. aeruginosa, S. aureus* and *S. epidermidis* biofilms and persisters	Banin et al., 2006; Chauhan et al., 2012
Fighting persisters	Inhibiting persister formation	The ppGpp analog, relacin, inhibited the *B. subtilis* RelA synthetase activity and biofilm formation	Wexselblatt et al., 2012, 2013
		Peptide 1018 displayed a specific antibiofilm activity against *P. aeruginosa* and *S. aureus* by inducing ppGpp degradation	de la Fuente-Núñez et al., 2014
		Art-175, an artilysin covalently combined a bacteriophage-encoded endolysin can pass the outer membrane to kill *P. aeruginosa* persisters	Briers et al., 2014
	Potentiating antimicrobials	Sugar metabolic stimuli potentiated aminoglycoside against *E. coli* and *S. aureus* persisters by facilitating aminoglycoside uptake	Allison et al., 2011
		Silver potentiated antimicrobials against bacterial biofilm and persisters by increasing ROS production and bacterial permeability to antimicrobials	Morones-Ramirez et al., 2013
		Raising pH by using basic amino acids (e.g., L-arginine) potentiated aminoglycosides activity against *E. coli* and *S. aureus* persisters	Lebeaux et al., 2014a
	Inactivating tolerance	AHL QS inhibitors, brominated furanones, could revert antimicrobial tolerance of *P. aeruginosa* and *E. coli* persisters	Pan and Ren, 2013
		Combining the acyldepsipeptide antibiotic that activates ClpP with rifampicin led to complete eradication of *S. aureus* biofilms	Conlon et al., 2013

The deep research on the mechanism of biofilm formation leads to the emergence of numerous promising antibiofilm approaches. However, the conversion of experimental data into clinical settings is time-consuming and to some extent unsatisfactory. Non-biocidal anti-adhesive or anti-virulence strategies face the diversity of bacterial phenotypes and may only be active against a subpopulation of bacteria encountered in clinical practice, therefore limiting their overall efficacy. *In vitro* biofilms are probably structurally different from *in vivo* biofilms (Lebeaux et al., 2013). Currently, due to the diversity of the *in vivo* conditions leading to the complexity of clinical biofilm situations, the diversity of persister phenotypes is unknown. Most of the studies use rodent models, but these *in vivo* models may not properly replicate real clinical state. Furthermore, as for clinical trials, rigorous statistical analysis is compulsory in order to avoid any false positive results. Most importantly, molecules identified *in vitro* should be validated using *in vivo* models not only for the antibiofilm activity but also non-toxicity.

## Metallic nanoparticles (NPs)

Physiochemical properties of nanomolecules as antimicrobial agents are widely used in human and veterinary medicine. Metallic NPs are of great interest for use as potential antimicrobial agents because of their unique optical, electronic, and magnetic properties (Kandi and Kandi, 2015). The electrostatic interaction of NPs with negatively charged bacterial surfaces draws the particles to the bacteria and promotes their penetration into the membrane, causing membrane disruption, bacterial flocculation, and a reduction in viability. The generation of reactive oxygen species (ROS) is also a mechanism of antibacterial activity of NPs (Thekkae Padil and Černík, 2013). Further actions of NPs as antimicrobial agents include disrupting DNA during the replication and cell division of microorganisms, compromising the bacterial membrane integrity via physical interactions with the microbial cell, and releasing toxic metal ions and causing lysis of cells (Franci et al., 2015). Recently, the silicon dioxide NPs (Si-NP) were engineered to target the signaling molecules (i.e., acylhomoserine lactones) used for QS in order to halt bacterial communication (Miller et al., 2015). The bactericidal activity of NPs depends on size, stability and concentration in the growth medium (Tillotson and Theriault, 2013). The applications of nanomolecules in medicine have recently been evaluated in reports highlighting the *in vitro* antimicrobial activities of NPs (Table 3), and also the possible potential adverse effects of nanomolecules on human health and the environment (Kandi and Kandi, 2015).

**Table 3:** **Selected studies on the antibacterial activity of metallic nanoparticles**.

**Nanoparticles**	**Target organisms**	**References**
Au-zeolites NPs	*E. coli* *Salmonella typhi*	Lima et al., 2013
PAH capped AuNPs and AgNPs	*Bacillus Calmette-Guérin* *E. coli*	Zhou et al., 2012
AgNPs	*Microcystis aeruginosa*	El-Sheekh and El-Kassas, 2014
AgNPs	*Enterococcus hirae*	Vardanyan et al., 2015
AgNPs/halloysite nanotubes/graphene nanocomposites (Ag/HNTs/rGO)	*E. coli* *S. aureus*	Yu et al., 2014
Selenium and AgNPs produced by *Bacillus*	*E. coli* *S. aureus* *Klebsiella* spp. *Pseudomonas* spp.	Singh et al., 2014
AgNO_3_ NPs produced by *Allium* sp	*E. coli* *Proteus* spp. *Klebsiella* spp. *Staphylococcus* spp. *Enterobacter* spp. *Bacillus* spp. *Pseudomonas* spp.	Lekshmi et al., 2012; Aramwit et al., 2014; El Kassas and Attia, 2014
ZnO NPs	*E. coli* *P. aeruginosa* *Aspergillus niger* *Salmonella choleraesuis* *S. aureus*	Azizi et al., 2013; Chitra and Annadurai, 2013
ZnO, CuO, and Fe_2_O_3_ NPs	*S. aureus* *B. subtilis* *P. aeruginosa* *E. coli*	Azam et al., 2012
Polyacrylamide-doped Fe_3_O_4_ NPs	*E. coli*	Mukherje, 2014
Ag-implanted Ti NPs	*Streptococcus mutans* *Porphyromonas gingivalis* *Candida albicans*	Zheng et al., 2012
H_2_TiO_3_ and SiO_2_ NPs	*E. coli* *S. aureus* *Enterococcus faecalis* *Candida* sp.	Krokowicz et al., 2015
Polymethyl methacrylate denture acrylic loading PtNPs	*S. mutans* *S. sobrinus*	Nam, 2014

As various metallic NPs and their oxides have already been used as potent antimicrobial agents, silver or ionic form is most toxic for microorganisms when compared to other metals (Seil and Webster, 2012). This makes silver of particularly interests. Silver NPs (AgNPs) probably have multiple mechanisms of antibacterial action (Markowska et al., 2013). For example, (1) AgNPs can affect bacterial membrane permeability by attaching to the cell membrane surface and modifying the cell potential; (2) AgNPs can causes oxidative damage by production of ROS (Kim et al., 2007; Xu et al., 2012); (3) AgNPs can interact with the SH-groups of bacterial membrane proteins and intracellular proteins, and also can interact with the phosphate residues in DNA, thus to interfere with protein synthesis and function and cell division (Durán et al., 2016). However, due to the current lack of knowledge, the exact basis for the activity of AgNPs is still uncharacterized.

The antibiofilm activity of AgNPs has also been demonstrated in a number of studies (Table 4). Most of the AgNPs are within the range of 1~100 nm. Although smaller AgNPs may have greater biological activity, it is important to note that differences in the chemical and physical properties of AgNP may cause variation in its antimicrobial and antibiofilm efficacy (Markowska et al., 2013). Because of the relatively low stability of colloidal solutions, some researchers propose the usage of stabilized AgNPs (Table 4). AgNPs can also enhance the antibacterial and antibiofilm activity of conventional antimicrobials. There are reports describing synergistic activity between AgNPs and, e.g., ampicillin, kanamycin, streptomycin or vancomycin against *E. coli* and *P. aeruginosa* (Wolska et al., 2012). Some AgNPs have been subjected to clinical trials (Franci et al., 2015).

**Table 4:** **Selected studies on the antibiofilm activity of AgNPs**.

**Diameter (nm)**	**Biofilm of microorganisms**	**References**
65 ± 30 nm	*P. putida*	Fabrega et al., 2009
50 nm	*P. aeruginosa* *S. epidermidis*	Kalishwaralal et al., 2010
25.2 ± 4 nm	*P. aeruginosa* (also kill the bacteria)	Martinez-Gutierrez et al., 2013
20~30 nm	Sensitive strain of *P. aeruginosa* (inhibition rate of 67%) Multidrug resistant strain of *P. aeruginosa* (inhibition rate of 56%)	Palanisamy et al., 2014
5~8 and 16~19 nm	*Salmonella enteritidis, S. hadar, and S. Senftenberg*	Losasso et al., 2014
12.6 ± 5.7 nm	*Mycobacterium* spp. (reduce survival of this bacterium to only 0.03%)	Islam et al., 2013
2.7 ± 0.6 nm (used for dental composites)	Oral bacteria *S. mutans*	Cheng et al., 2012
47 nm (citrate-capped)	*P. aeruginosa*	Park et al., 2013
20 nm (silver coated polyvinyl pyrrolidone)	*Streptococcus pneumoniae*	Bibbs et al., 2014
20 nm (starch-stabilized)	*P. aeruginosa* *S. aureus*	Mohanty et al., 2012
10 nm (used to coat the surface of catheters)	(complete inhibition) *E. coli* *S. aureus* *Candida albicans* (more than 50% inhibition) *Enterococcus* sp. coagulase-negative staphylococci *P. aeruginosa*	Roe et al., 2008
8 nm (hydrolyzed casein peptide- stabilized)	*E. coli* *P. Aeruginosa* *Serratia proteamaculans*	Radzig et al., 2013
4–7 nm (β-cyclodextrin-stabilized)	*S. epidermidis CSF 41498*	Jaiswal et al., 2015
1.9~4.3 nm (microwave accelerated and *Eucalyptus globulus* leaf extract-stabilized)	*S. aureus (MRSA)* S. *aureus* (MSSA) *P. aeruginosa (ESBL)*	Ali et al., 2015

Although metallic NPs have great potential in the future to become antimicrobial agents, the local and systemic toxic complications and the deleterious effect they have on beneficial bacteria in humans and animals may be a cause for concern (Zhang et al., 2010; Prabhu and Poulose, 2012). NPs have the ability to spread throughout the body, cross the blood-brain barrier, cause haemolysis, and may result in degradation products which have toxic effects and influence blood coagulation pathways (Kandi and Kandi, 2015). Most studies have not conclusively evaluated the exact mechanism by which nanomolecules contribute to toxic complications, and many of the interactions of the AgNPs with the human or animal body are still poorly understood (dos Santos et al., 2013). It has been observed that the larger size of NP, the greater is the risk of adverse health effects (De Jong and Borm, 2008). Research is necessary to clearly understand the interaction of nanomolecules with living cells, the extent of their distribution in the body, and their specific organ toxicity.

## Concluding remarks

The paradox of antimicrobial drugs is that through their use, they not only inhibit an infection but also select for the emergence and spread of AMR, directly counteracting their long-term efficacy. Considerable inappropriate use of both prophylactic and therapeutic antimicrobials in human and veterinary medicine highlights an urgent need for antibiotic stewardship initiatives. At the present time, rational use of existing antibiotics based on PK/PD dosage-regimen is a key strategy in tackling AMR, thus to preserve potent antimicrobials for both humans and animals. At the same time, we should never stop discovering novel inhibitors with new bacterial targets and digging the treasure box of “old” and “forgotten” antimicrobials. Compounds showing profound anti-resistance and antibiofilm effects are in research hotspot, but they still have limitations. Combining existing antimicrobials with compounds that inhibit their specific resistance mechanisms would be a good choice. Although metallic NPs can both kill bacteria and inhibit biofilm formation, the toxicity is still a big challenge for their clinical applications (dos Santos et al., 2013). With single-drug therapy, there is always a selective advantage to resistance; specific combinations of drugs can inhibit bacterial growth while disfavoring resistance to the individual components. These approaches can be used to invert the selective advantage of resistant bacteria competing with their sensitive cousins, or even drive a resistant bacterial population back toward drug sensitivity (Baym et al., 2016). Besides, screening and developing multiple-target inhibitors as “resistance-resistant” antimicrobials can reduce the effects of target mutation (Oldfield and Feng, 2014). The natural products have also been a prolific and unsurpassed source for new lead antimicrobial compounds, but target identification and validation has remained a major bottleneck (Farha and Brown, 2016). Functional genomics techniques are proved to be indispensible for *in vitro* target authentication and elucidating mechanism of action of novel antimicrobials (Khan and Khan, 2016). Since most of the new strategies described in this review are only at the early basic experimental stage, their potential for clinical applications requires more extensive investigations.

## Author contributions

GC contributed to the design of the review and wrote the review. MD, SA, HH, and XW revised the review. ZY contributed to the conception of the review.

### Conflict of interest statement

The authors declare that the research was conducted in the absence of any commercial or financial relationships that could be construed as a potential conflict of interest.

## Abbreviations

AMR: antimicrobial resistance
HGT: horizontal gene transfer
MDR: multidrug-resistant
MIC: minimum inhibitory concentration
NPs: nanoparticles
PK: pharmacokinetic
PD: pharmacodynamic
QS: quorum sensing
ROS: reactive oxygen species.
